# Ten-year trend analysis of malaria prevalence in Gindabarat district, West Shawa Zone, Oromia Regional State, Western Ethiopia

**DOI:** 10.1186/s12936-024-04975-2

**Published:** 2024-05-16

**Authors:** Kinfu Boresa, Tariku Belay, Abdissa Biruksew, Eshetu Alemayehu, Endalew Zemene

**Affiliations:** 1 Department of Medical Laboratory Sciences, Institute of Health Sciences, Wallaga University, Nekemte, Ethiopia; 2 School of Medical Laboratory Sciences, Institute of Health, Jimma University, Jimma, Ethiopia; 3 Department of Epidemiology and Biostatistics, Institute of Health, Jimma University, Jimma, Ethiopia

**Keywords:** Malaria, Prevalence, Retrospective, Gindabarat, Ethiopia

## Abstract

**Background:**

Malaria is a major public health concern in Ethiopia, where more than half of the population lives in malaria risk areas. While several studies have been conducted in different eco-epidemiological settings in Ethiopia, there is a notable scarcity of data on the prevalence of malaria in the Gindabarat district. Therefore, this study aimed to analyse 10-year trend of malaria prevalence in Gindabarat district, West Shawa Zone of Oromia, Western Ethiopia.

**Methods:**

A retrospective laboratory record review was conducted at Gindabarat General Hospital and Gindabarat District Health Office from September 2011 to August 2020. The retrieved data included the date of examination, age, sex and laboratory results of the blood smears, including the *Plasmodium* species identified. Data were summarized and presented in the form of tables, figures, and frequencies to present the results. The data were analysed using SPSS (version 25.0) and Microsoft Excel.

**Results:**

Over the course of 10 years, a total of 11,478 blood smears were examined in the public health facilities in the district. Of the total blood smears examined, 1372 (11.95%) were microscopically confirmed malaria. *Plasmodium falciparum*, *Plasmodium vivax* and mixed infections (*P. falciparum* and *P. vivax*) accounted for 70.77%, 20.55% and 8.67% of the cases, respectively. Malaria prevalence was significantly higher among individuals aged ≥ 15 years (12.60%, x^2^ = 13.6, df = 2, p = 0.001) and males (14.21%, x^2^ = 59.7, df = 1, p = 0.001). The highest number of malaria cases was recorded from September to November.

**Conclusion:**

Malaria remains a public health problem in the district. *P. falciparum* was the most predominant parasite species in the area. Malaria prevalence was significantly higher among individuals aged ≥ 15 years and males. There was a remarkable fluctuation in the number of malaria cases in different months and years. In the study area malaria cases peaked in 2015 and 2017 then decreasing from 2017 to 2019, with sharp increase in 2020. Moreover, this study showed malaria cases were reported in all seasons and months, but the highest was observed from September to November. Strengthening malaria control activities is essential to further reduce the burden of malaria and pave the way for the anticipated elimination.

**Supplementary Information:**

The online version contains supplementary material available at 10.1186/s12936-024-04975-2.

## Background

Malaria, while preventable and treatable, is one of the world's deadliest diseases, and has a substantial influence on public health and economic development of tropical countries. In 2022, an estimated 249 million malaria-related illnesses and 608,000 deaths were reported globally [1]. Sub-Saharan Africa bears a disproportionately high burden of the disease, and children under 5 years of age are particularly vulnerable. Over the last two decades, remarkable achievements have been obtained in malaria control globally. Several countries have been planning for malaria elimination, some of which have already eliminated malaria from their national territories in the past two decades [2]. Nonetheless, the successes obtained in the control of malaria are threatened by insecticide resistance in malaria vectors, anti-malarial resistance in malaria parasites and the occurrence of invasive malaria vectors [3–5].

Despite the considerable gains in the control of malaria, it remains a public health problem in Ethiopia. Approximately three-quarters of the land mass is favorable for malaria transmission, where more than half of the population lives. In most malaria risk areas of the country, malaria transmission is seasonal, and peaks between September and December following the major rainy season, which spans from June to August. Areas below 1000 m above sea level, mainly the western lowlands, experience perennial high burden malaria transmission. Almost all cases of malaria in Ethiopia are caused by *Plasmodium falciparum* and *Plasmodium vivax,* and there is remarkable spatiotemporal variation in the distribution of these species [6, 7]. Malaria in Ethiopia is primarily transmitted by *Anopheles arabiensis*, while *Anopheles pharoensis*, *Anopheles funestus* and *Anopheles nili* play a secondary role. Of concern is the recent detection and distribution of the invasive malaria vector *Anopheles stephensi* in Ethiopia [8, 9], and other Eastern African countries [10, 11]. *Anopheles stephensi* is native to parts of Asia, and was found to have been naturally infected with *Plasmodium* and implicated in urban malaria outbreaks in eastern Ethiopia [12]. Malaria control in Ethiopia mainly involves the deployment of the core vector interventions (insecticide-treated nets [ITNs] and indoor residual spraying [IRS]) and passive case detection and treatment of cases.

Analysing the trend of malaria in a particular setting is essential for better understanding of the effectiveness of control interventions and progress towards elimination. Ethiopia has planned an ambitious goal of eliminating malaria by 2030. Nevertheless, there have been reports of malaria outbreak in several areas in Ethiopia in recent years [13–15]. According to the 2023 World Health Organization (WHO) malaria report, the number of malaria cases in Ethiopia increased by 1.3 million in 2022 compared to the preceding year [1]. Despite favourable climatic conditions for malaria transmission in Gindabarat district and recent challenges related to disruption of the health care system due to COVID-19 pandemic, the trend of malaria cases in the district is not known. The aim of this study was to describe the trend of malaria cases diagnosed at Gindabarat General Hospital and the district health office, West Shawa Zone, Western Ethiopia.

## Methods

### Study setting

**Fig. 1:**
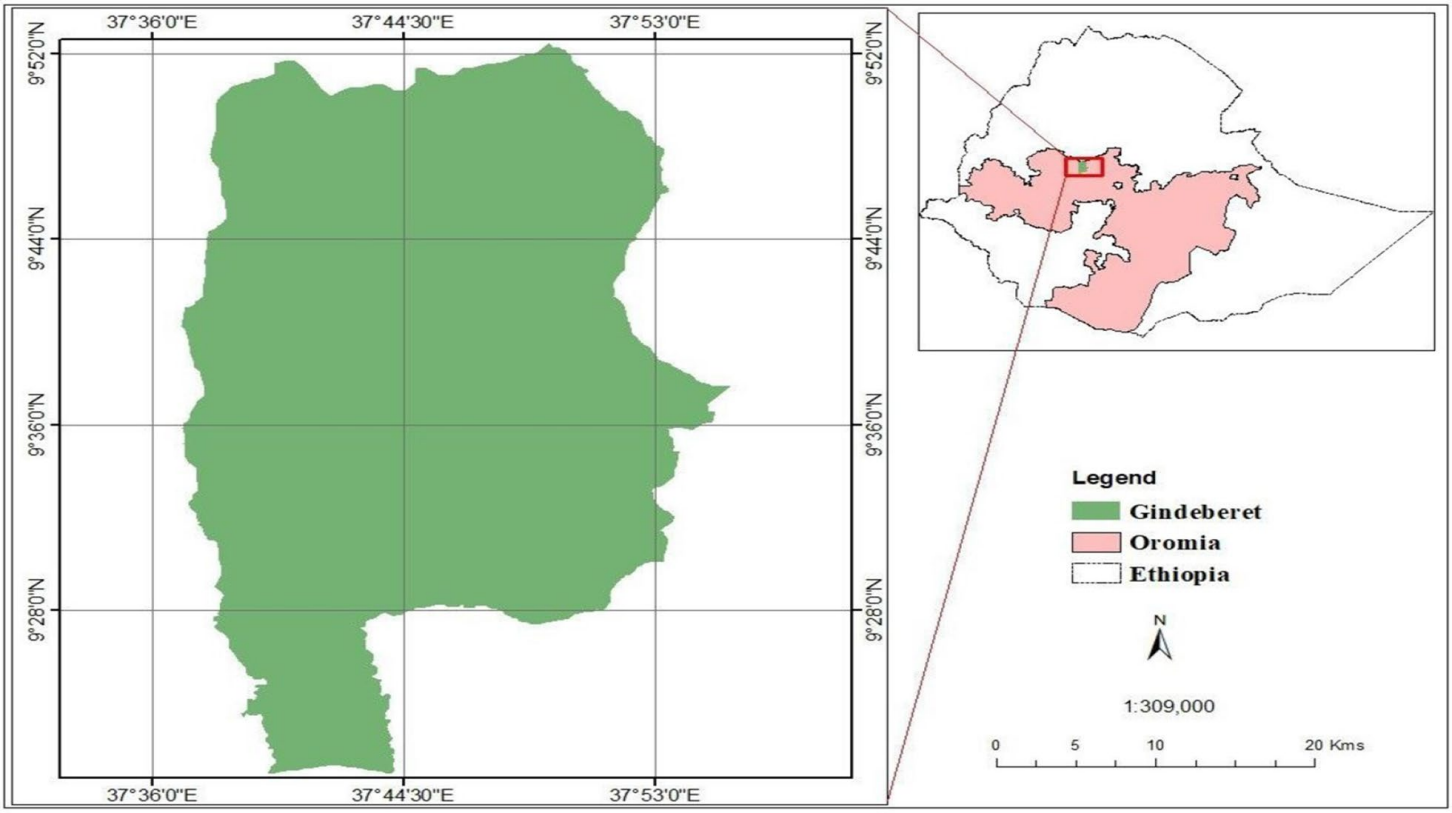
Map of the study area

The study was conducted in Gindabarat district, located in the West Shawa Zone, Oromia Regional State, Western Ethiopia. The district is 200 km from Addis Ababa (Fig. 1). Gindabarat District has an altitude range of 1500–3500 m above sea level and is characterized by a mean annual temperature ranging from 20 to 25 °C [16, 17], and an average annual rainfall is 1150 mm. According to the projection of the Central Statistical Agency of Ethiopia carried out in 2015, the estimated population of the district was 104,595 people, 52,726 (50.4%) of whom were male and the remaining 51,869 (49.59%) were female [18]. The district has 32 health posts, six health centres and one General Hospital. Gindabarat General Hospital and the district health offices were selected for this study since health posts are almost all newly established health institutions and most of the district population visits Gindabarat General Hospital and district health offices (health centres monthly report a carbon copy which used as a logbook to the district health office). Malaria is the most prevalent and seasonal disease in areas where both *P. vivax* and *P. falciparum* co-exist.

### Study design

Institution-based retrospective cross-sectional study was conducted by reviewing the malaria case records from registers of district health office and Gindabarat General Hospital from 01 September to 30 December 2021.

### Study population

The study population included all individuals with suspected malaria who had visited Gindabarat General Hospital, and district health offices (monthly report from health centres of the district) from September 2011 to August 2020. The study covered the period from 2011 to 2020, capturing data on all confirmed malaria cases diagnosed and treated at the hospital that fulfilled the inclusion criteria.

### Inclusion criteria

The analysis included data such as the number of malaria cases diagnosed in months and years, the types of malaria species identified, and sociodemographic data (age and sex) regardless of pregnancy, or other infection status.

### Exclusion criteria

Any data that did not meet the inclusion criteria were excluded. Data with incomplete information on any of the relevant variables  were excluded. The data included year and month of the visit, sex, age, status of the blood film (positive or negative), and species of *Plasmodium* detected.

### Sample size

All 11, 478 malaria reports documented in the laboratory logbooks and reports of the health centres from September 2011 to August 2020 that met the inclusion criteria were taken for analysis.

### Data collection and quality control

A well-organized checklist was used for collecting data on malaria cases and related information registered from 2011 to 2020. The data were retrieved from the laboratory logbooks of Gindabarat General Hospital and the district health office. The information contained on the checklist included the year and month of the visit, sex, age, status of the blood film (positive or negative), and species of *Plasmodium* found. An expert medical laboratory technologist collected the data. The WHO protocol was followed in the hospital, where microscopic blood film screening was performed as the gold standard to confirm the presence of *Plasmodium* parasites and identify the species. The national standard operating procedure was followed for examining blood films for malaria parasites. The microscopic examination was carried out by laboratory technologists or technicians who had received thorough training in the microscopy of malaria. Throughout the 2011–2020 study periods, microscopy was the only method employed to identify *Plasmodium* species because there is expertise and electricity in all health facilities (health centres and district hospital). The data were gathered under supervision, and before analysis, they were verified as comprehensive. Prior to the study, data collectors and supervisor were trained for 2 days to insure the quality of data. They were trained on the data collection tools, variables of interest, rationale, objective and significance of the study. Similarly, the data entry clerks were trained on the same points. The whole process, data capturing and data entry was daily supervised by principal investigator to ensure the completeness and consistency of the data. Data that were not fully registered were not included in the analysis.

### Data processing and analysis

To conduct the analysis, the obtained data were input into Epidata version 3.1 and exported Statistical Package for Social Sciences (SPSS) version 25. Some of the figures were also created using Microsoft Excel. Tables, figures, and frequencies were used to present the results. To ascertain the frequencies and percentages of general malaria prevalence and trend prevalence in terms of year, season, *Plasmodium* species, sex, and age, descriptive statistics were used. The correlation between malaria burden, and sex and age group was examined using a chi-square test. A P-value of 0.05 or lower indicated statistical significance.

## Results

During the 10 years (2011–2020), a total of 11,478 blood films from malaria-suspected patients were diagnosed at Gindabarat General Hospital and district health office. The prevalence of malaria fluctuated during the 10 years of the study with a minimum (8.4%) and maximum (13.5%) annual malaria prevalence reported in 2019 and 2017, respectively, as indicated below. The number of suspected malaria cases peaked between 2011/12 and 2018 in the district (Table 1).

**Table 1:** Annual trends in total malaria cases in Gindabarat General Hospital and district health office, Western Ethiopia; from 2011 to 2020

Year	Blood films examined	Laboratory-confirmed malaria cases	Slide positivity rate (percent)	*Plasmodium* species		
				*P. falciparum*	*P. vivax*	Mixed (*Pf* and *Pv*)
2011	1861	240	12.95	190	33	17
2012	1070	133	12.33	100	24	9
2013	1003	112	11.16	76	25	11
2014	826	103	12.59	68	23	12
2015	638	86	13.47	61	17	8
2016	877	113	12.65	76	27	10
2017	947	127	13.5	83	32	12
2018	1581	185	11.7	128	37	20
2019	1445	122	8.4	83	29	10
2020	1230	151	12.27	106	35	10
Total	11,478	1372	11.95	971	282	119

**Fig. 2:**
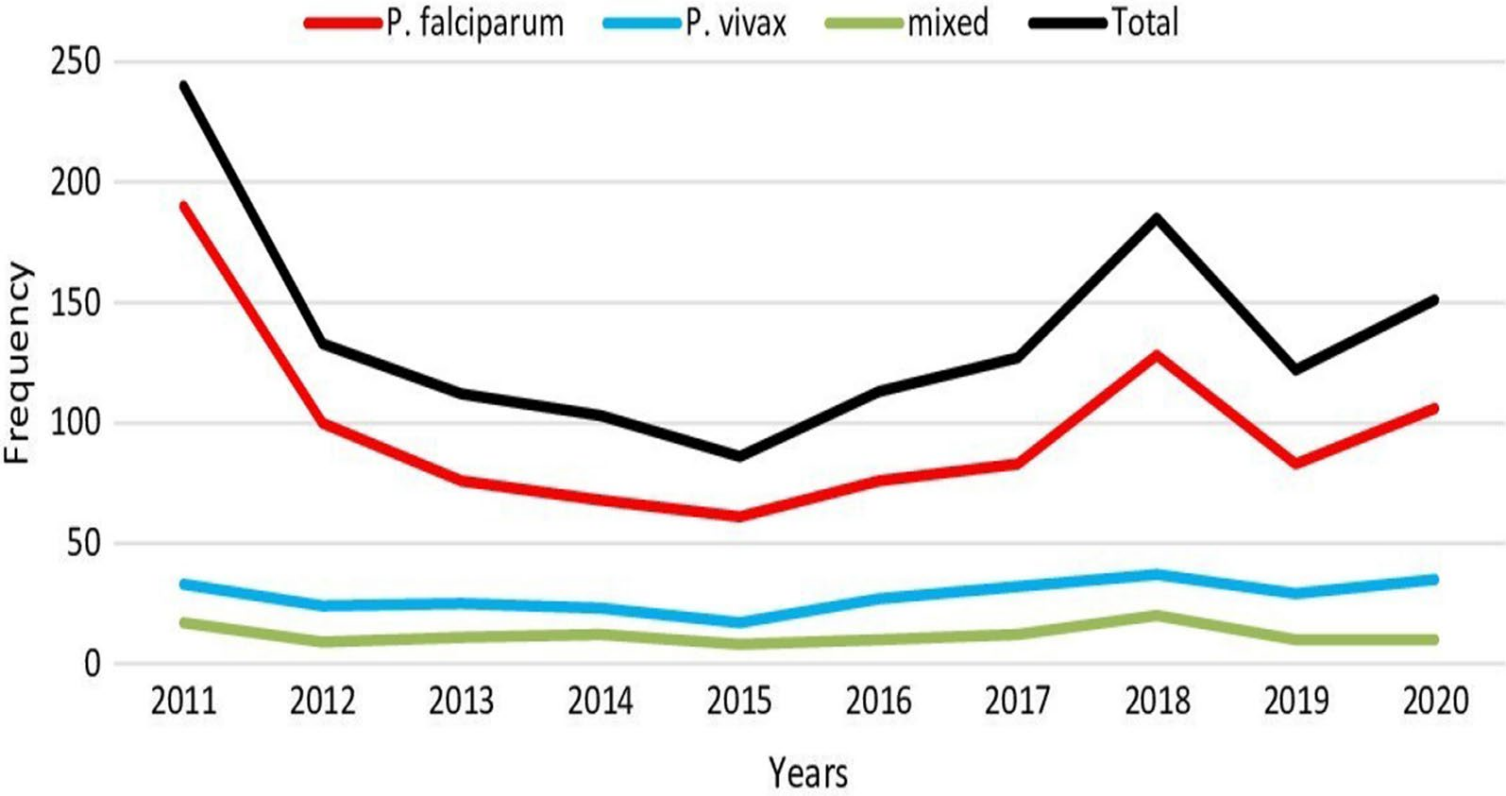
Distribution of malaria cases by diagnosis years at Gindabarat General Hospital and district health office, Western Ethiopia from 2011 to 2020

Figure 2 shows that the number of *P. falciparum*, *P. vivax* and mixed infections increased from 2014 to 2018. There were 6 *P. vivax* cases and 23 *P. falciparum* cases, both increasing from 2019 to 2020.

**Fig. 3:**
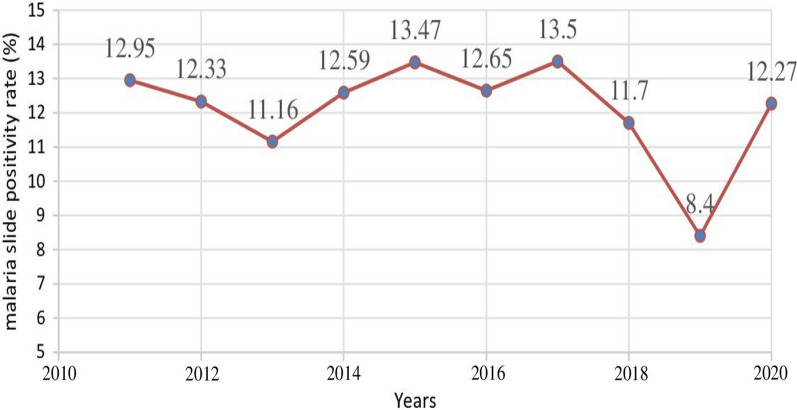
Blood smear-positive rate of malaria in Gindabarat district, Western Ethiopia, from 2011 to 2020

As indicated by Fig. 3, the high slide positivity rate (SPR) of the malaria parasite indicates a high prevalence of malaria among patients (a high rate of malaria infection). The slide positivity rate started to decline between 2011 and 2013, 2015 and 2016, and 2017 and 2019, with the lowest number of cases reported in 2019(8.4%). However, the slide positivity rate increased between 2013 and 2015, 2016 and 2017, and 2019 and 2020, with the highest number of positive results reported in 2017 (13.5%).

Generally, there was a fluctuation in the number of malaria cases throughout the study period, and the graphs of the yearly number of malaria cases exhibited a “V” shape from 2011 to 2015, 2015 to 2017, and 2017 to 2020 (Fig. 3).

Graphical representations of the significance checks of the mean differences between malaria cases within a year over a timeline were checked by using one way ANOVA post hoc Tukey’s test, as indicated by the output below. There was a statistically significant difference in the means between and within years from 2011 to 2020 (F (9, 11,468) = 2.698, P = 0.004). Generally, there is a statistically significant mean difference among years from 2011 to 2020, as illustrated in the ANOVA output (Table 2).

**Table 2:** One way ANOVA post hoc Tukey’s test, for significance checks of the mean difference in Gindabarat district, Western Ethiopia, from 2011 to 2020

Result	ANOVA				
	Sum of squares	df	Mean square	F	Sig.
Between groups	2.559	9	0.284	2.698	0.004
Within groups	1206.967	11,468	0.105		
Total	1209.522	11,477			

**Fig. 4:**
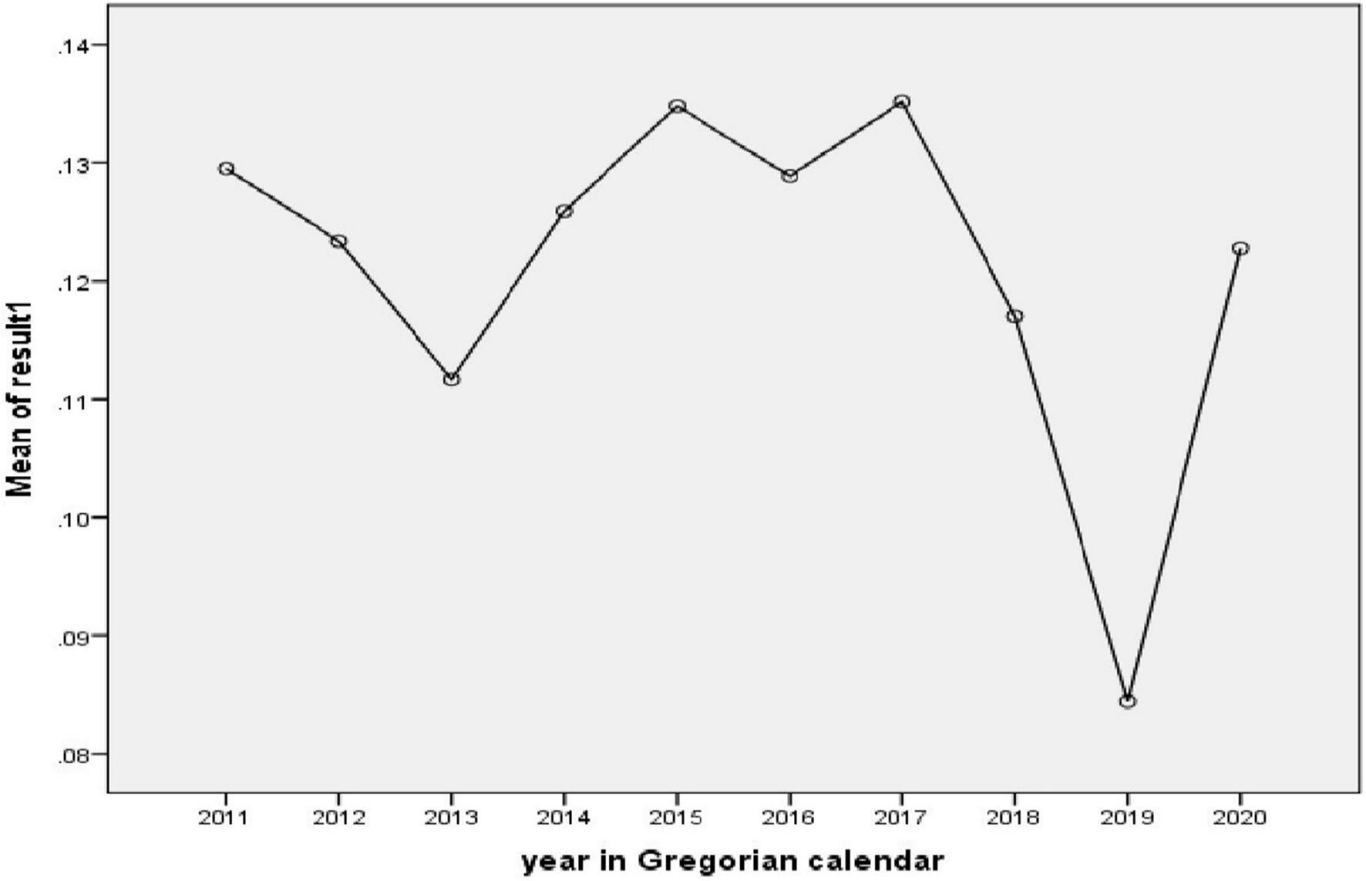
Mean difference plots for malaria cases within and between 2011 and 2020. The mean difference significantly decreased from 2011 to 2019 (95% CI = 0.0091–0.811; P = 0.003), 2015 to 2019 (95% CI = 0.0016–0.992; P = 0.037), 2016 to 2019 (95% CI = 0.0005–0.884; P = 0.045) and 2017 to 2019 (95% CI = 0.0078–0.0937; 0.007). However, from 2019 to 2020, the mean difference significantly increased (95% CI = 0.0015–0.0782; P = 0.007)

The mean difference in malaria cases significantly decreased from 2011 to 2019, 2015 to 2019, 2016 to 2019, and from 2017 to 2019. However, the mean difference in malaria cases from 2019 to 2020 significantly increased (Table 3; Fig. 4).

**Table 3:** Malaria case mean difference significance checks in Gindabarat district, Western Ethiopia from 2011 to 2020

Multiple comparisons						
Dependent variable: malaria result Tukey HSD						
(I) year during the diagnosis took place	(J) year during the diagnosis took place	Mean difference (I–J)	Std. error	Sig.	95% confidence interval	
					Lower bound	Upper bound
2011	2019	0.04507^a^	0.01137	0.003	0.0091	0.0811
2015	2019	0.05037^a^	0.01542	0.037	0.0016	0.0992
2016	2019	0.04442^a^	0.01389	0.045	0.0005	0.0884
2017	2019	0.05073^a^	0.01356	0.007	0.0078	0.0937
2019	2020	0.03834^a^	0.01259	0.0071	0.0015	0.0782

^a^Indicates that the difference is significant at the 0.05 level

### Distribution of malaria cases by sex

**Fig. 5:**
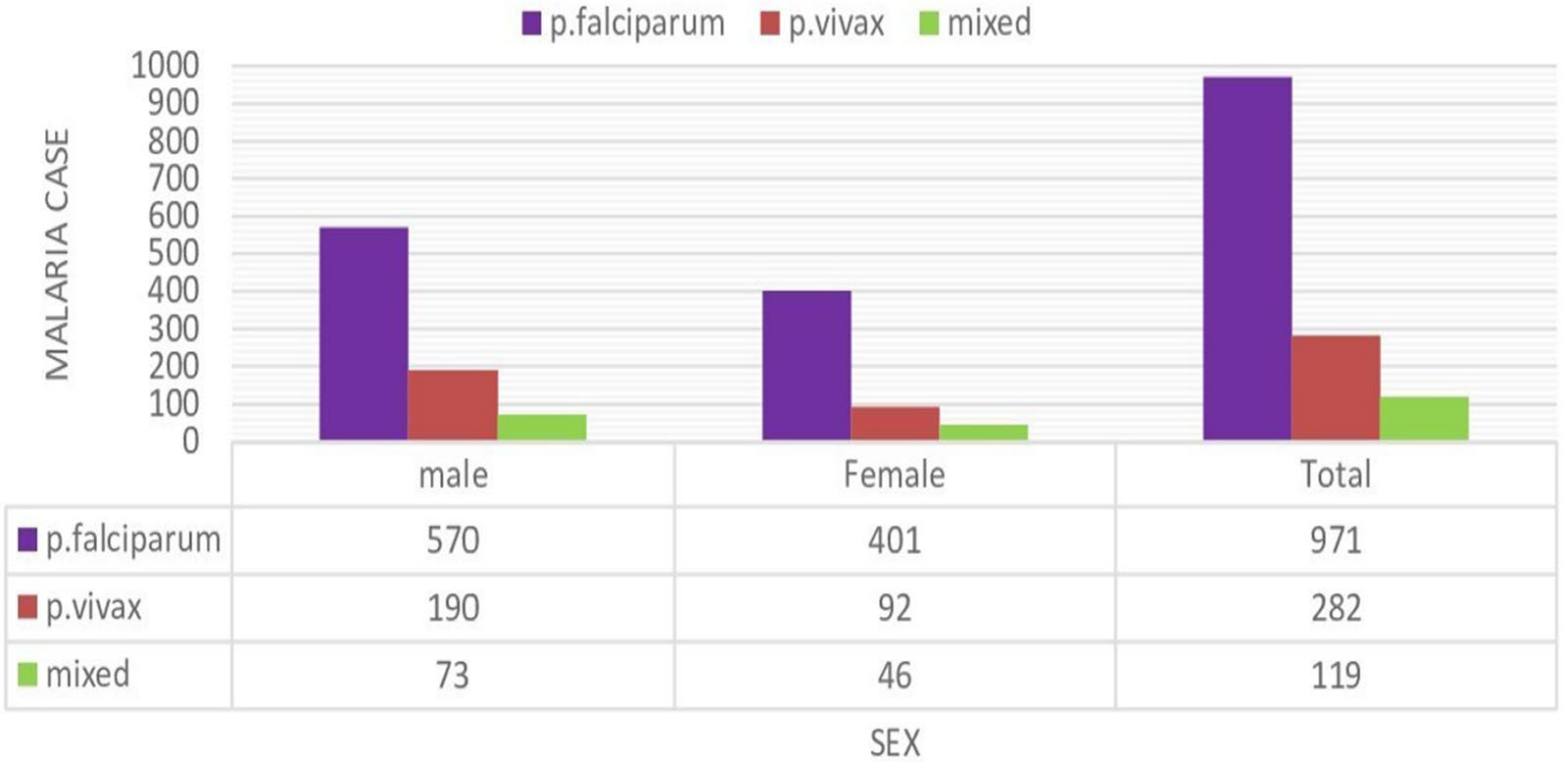
Distribution of malaria infection by sex in Gindabarat district, Western Ethiopia, from 2011 to 2020

Of the 1372 confirmed malaria cases, 14.21% (833/5862) were reported among males, while the remaining 9.60% (539/5616) were reported among females, with a male-to-female ratio of 1.54. The distribution of *Plasmodium* species in relation to sex is shown in Fig. 5. There was a statistically significant association between malaria prevalence and sex (x^2^ = 59.7, df = 1, P = 0.001) (Fig. 5).

### Distribution of *Plasmodium* species by age

**Fig. 6:**
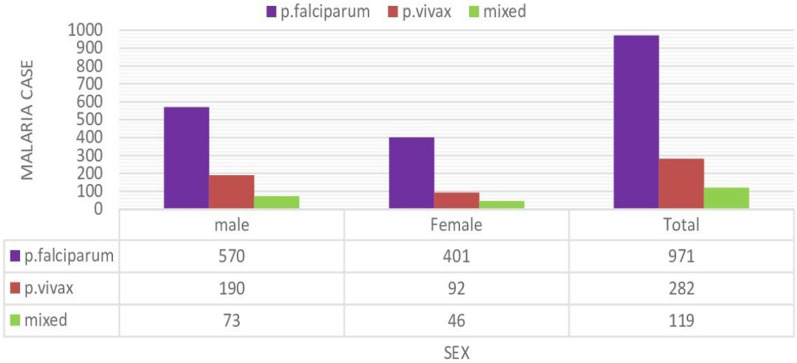
Distribution of malaria cases by age group in Gindabarat General Hospital and district health office, Western Ethiopia from 2011 to 2020

The distribution of parasite species in relation to the age group is shown in Fig. 6 above. There was a statistically significant association between malaria prevalence and age group (x^2^ = 13.6, df = 2, P = 0.001). Patients in the ≥ 15 years age group were more affected, with a prevalence rate of 12.60% (1044/8284), followed by those in the 5–14 years age group and under-five years of age, with prevalence rates of 10.65% (251/2356), and 9.20% (77/837), respectively. Concerning *Plasmodium* species, *P. falciparum* was the predominant species in all age groups, and was more common in the ≥ 15 years age group; moreover, *P. vivax* was the 2nd dominant species in the same age groups, with a prevalence rate of 727 (52.98%) and 221 (16.1%) (Fig. 6).

### Seasonal distribution of malaria

**Fig. 7:**
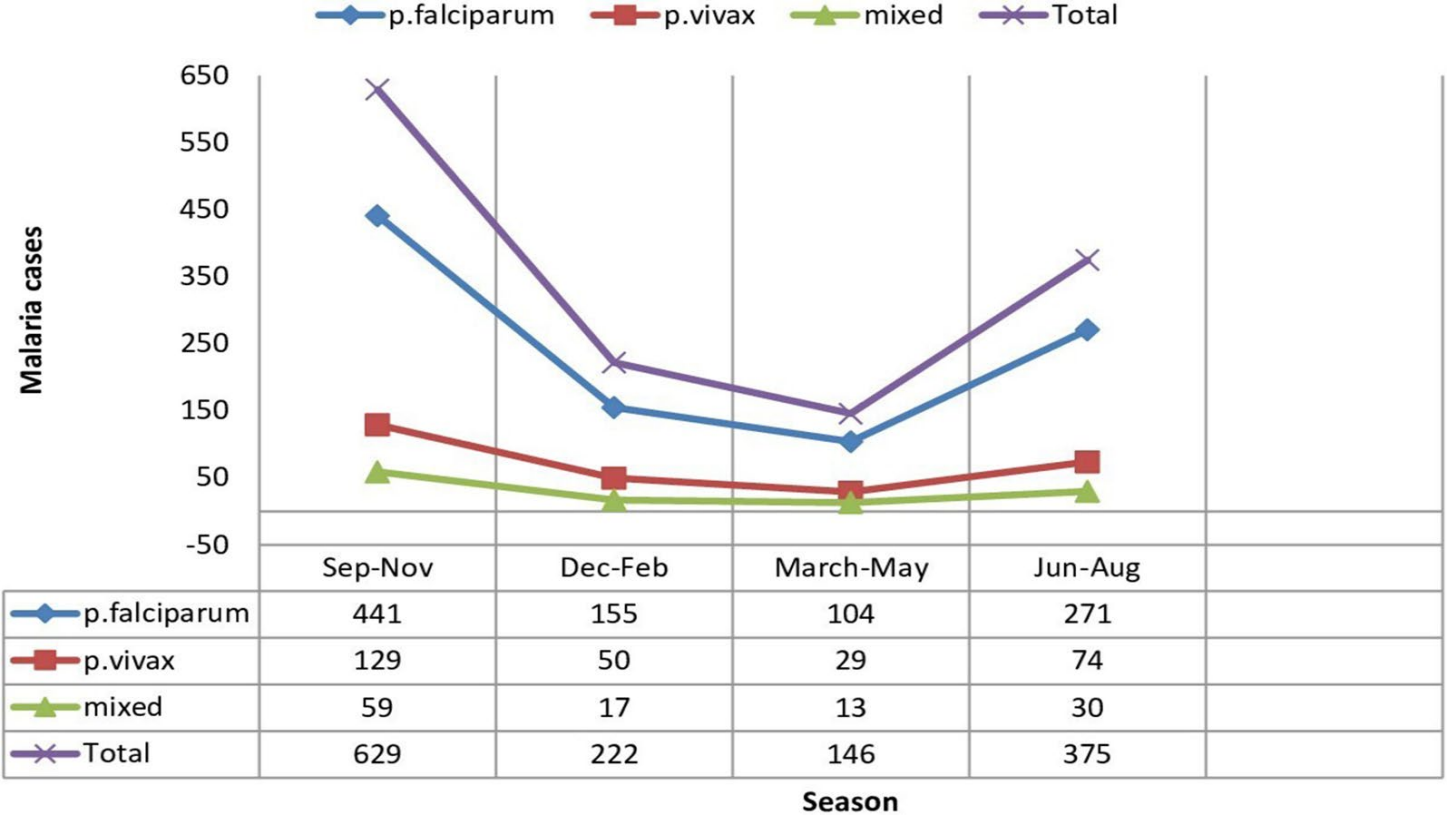
Distribution of *Plasmodium* species in different seasons in the Gindabarat district from 2011 to 2020, Western Ethiopia

Malaria cases have been reported in all seasons and months. The outcome of this study showed that the highest number of malaria cases was observed from September to November and the lowest was registered from March to May. The study also showed the species levels side by side, the maximum number of *P. falciparum* was registered in all seasons followed by *P. vivax*, and a minimum number of mixed infections (*P. falciparum* + *P. vivax*) were recorded in all seasons when compared to each other (Fig. 7).

## Discussion

Malaria is a major public health concern in many regions of the world, especially in sub-Saharan Africa. Ethiopia is one of the most common forms of infection in this region. Patients in this region suffer greatly from malaria, which has high transmission rates and related morbidity and mortality. The slide positivity rate in the retrospective study was 11.95%. The slide positivity rate in the retrospective study was 11.95%. This finding is comparable with the finding of other studies conducted in Ethiopia such as those of Arsi Negele (11.4%) [19], Kombolicha (7.52%) [20] and Northern Shoa (8.4%) [21]. In contrast, the prevalence in the present study was much greater than that in a study conducted in South Arabia (0.1%) [22]. However, the current malaria prevalence is much lower than that reported in studies conducted in Wereta town (32.6%) [23], Kola Diba (39.6%) [24], the Welega zone (20.07%) [25], and the Omo Zone of Southern Ethiopia (41.5%) [26]. These differences in malaria prevalence might be due to differences in climatic conditions, the skill of laboratory personnel in identifying malaria, the types of malaria intervention activities in the areas, and the diagnostic techniques used for malaria. The number of malaria cases is increasing in various parts of Ethiopia as a result of malaria outbreaks or high malaria transmission settings.

The prevalence of malaria has fluctuated annually, with the maximum and minimum numbers of cases recorded in 2017 and 2019, respectively. The decrease in malaria cases in 2019 might be comparable to the increase in awareness of the community toward the application of ITNs and the minimization of environmental compliance. This is because Ethiopia currently plans to eliminate malaria by 2030 in collaboration with different stakeholders so that the community has a strong willingness to anticipate (prevention) and initiate malaria control over the past decade. This result was in line with the global decreasing burden of malaria, which between 2000 and 2019 resulted in the averting of 1.5 billion cases; the majority of these instances (82%) were in the WHO’s African areas, which includes Ethiopia [27]. There has also been a notable drop in malaria recorded in Ethiopia [28–30].

In this study, the prevalence of malaria was higher in males [14.21%, (833/5862)] than in females [9.60%, (539/5616)], which is comparable with earlier studies carried out in Eastern Wollega [25], Southwest Ethiopia [31] and South central Ethiopia [32]. This might be due to the occupation of males and their lifestyle. Males are usually involved in irrigation activities, agricultural activities, and day labour, which might be suitable environments for mosquito breeding sites; alternatively, males are usually engaged in outdoor activities at dusks and dawns, which may coincide 16 h after peak biting. These findings are in line with those of a study conducted in India [33]. Among the age groups, the prevalence of malaria was highest in the 15 years and above age group (12.60%), followed by the 5–14 years age group (10.65%). This was in agreement with the findings of a study conducted at the Kola Diba Health Centre [24]. However, in contrast to these findings, a study conducted in Wolaita Zone showed a high malaria positivity rate in 5–14 year-olds [34]. The reason why malaria affects individuals aged 15 years and older in the Gindabarat district might be because these age groups are productive and, as a result, are actively involved in irrigation and agricultural activities; this can increase the exposure of these groups of people to *Anopheles* mosquito bites.

In the study area, the number of malaria cases peaked from September to November, which was in agreement with the findings in Dembia [35], Dembecha [36], Northwest Tigray [37], Ataye [21], Guba [38], Jimma [30], and Harari [39], followed by June to August. In seasonal transmission areas in Ethiopia, malaria cases usually peak from September to November following the major rainy season, which spans from June to August, creating a suitable environment for the breeding of *Anopheles* mosquitoes. The minor transmission period is a result of a small amount of rain from April to May. This discrepancy may be the result of the presence of advantageous local conditions, such as standing water and host spots in the microenvironment after a substantial rainfall followed by a dry season. These conditions foster an environment that is favourable for the growth of the mosquito population, the survival of the parasite in the mosquito, the rate of bites, and the spread of malaria parasites.

There was a significant yearly fluctuation in the number of malaria cases throughout the study period. Four significant peaks were observed in 2011, 2015, 2017, and 2020. The findings of our study revealed that there was a fluctuating trend in the occurrence of malaria in the study area over the course of 10 years. A significant decrease in the number of malaria cases occurred between 2011 and 2019, 2015 and 2019, and 2017 and 2019, with a minimum number of malaria cases reported in 2019 (8.4%). Malaria control interventions have generally intensified in Ethiopia over last decade, with the goal of eliminating malaria by 2030. However, there was a significant increase in the number of malaria cases from 2019 to 2020, with the peak number of malaria cases being reported in 2017(13.5%). The significant increase in malaria cases from 2019 to 2020 might be due to the COVID-19 pandemic, which might have interrupted health services.

The mean difference in malaria cases within and between years in the research area fell from 2011 to 2013, but increased in the following 2 years (2014 and 2015). The malaria cases were at the highest peak in 2015 and 2017 then it was decreasing from 2017 to 2019, with sharp increasing 2020. The decrease in malaria incidence between 2011 and 2013 and between 2017 and 2019 may be related to community awareness raising about the use of various insecticides and repellents (such as buzz of), enhancing indoor residual spray operation quality, and reducing environmental compliance.

Malaria cases have fluctuated in general during the last 10 years in the study area. Many factors, including host and vector characteristics, social and economic influences, and changes in healthcare infrastructure, could contribute to these fluctuations. Mosquito control measures, population immunity, government policy, availability of health facilities, and drug resistance, 17 among other social, biological, and economic factors, all have significant impacts on malaria prevalence [40, 41].

## Limitations of the study

The primary limitation of this study was the inconsistency and incompleteness of the retrospective data because certain essential variables such as sociodemographic data (age and sex) were missing.

## Conclusion and recommendation

The total malaria prevalence declined in the study area, indicating good progress toward meeting the 2030 malaria elimination goals. Malaria, however, remains a public health concern in the region, affecting 11.9% of the population. *P. falciparum* is the prevalent species in the research region, indicating a change from *P. vivax* to *P. falciparum* malaria, which puts the ongoing malaria elimination campaign at risk. The reproductive age group and males were more afflicted by the infection, which was more prevalent/common during the cultivation season, affecting public health and economic development of the area. Therefore, malaria control and elimination programs should be strengthened to further reduce the burden of malaria, particularly among highly affected groups. There is also a need to intensify the prevention and control strategies for *P. falciparum* in this area.

### Supplementary Information

Supplementary Material 1.

## Data Availability

All the data are available from the corresponding author upon reasonable request. All relevant data are within the manuscript.

## Abbreviations

IRS: Indoor residual spraying
ITNs: Insecticide-treated nets
SPSS: Statistical Package for the Social Science
WHO: World Health Organization
COVID-19: Corona virus disease of 2019
ANOVA: Analysis of variance
SPR: Slide positivity rate
