# Supplementary figures and images for: Ten-year trend analysis of malaria prevalence in Gindabarat district, West Shawa Zone, Oromia Regional State, Western Ethiopia
Source: Malar J. 2024 May 16;23:152. doi: 10.1186/s12936-024-04975-2 (PMC11100101; doi:10.1186/s12936-024-04975-2)

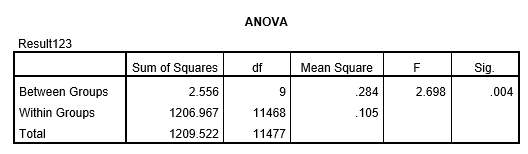


ANOVA result that shows between and within groups

Supplement: Supplementary file 1 — Supplementary Material 1. [file 12936_2024_4975_MOESM1_ESM.docx]
